# Supplementary material for: Eye Movement Evaluation in Multiple Sclerosis and Parkinson's Disease Using a Standardized Oculomotor and Neuro-Ophthalmic Disorder Assessment (SONDA)
Source: Front Neurol. 2020 Sep 8;11:971. doi: 10.3389/fneur.2020.00971 (PMC7506055; doi:10.3389/fneur.2020.00971)
Supplement: Supplementary file 1 [file Data_Sheet_1.PDF]

Supplementary material

1 Spatio-temporal properties of eye movements: description of algorithms and features

This supplementary material describes the stimulus, algorithms and resulting features that are used in the spatio-temporal analysis of the properties of eye movements. The main algorithm described in this chapter is partially based on the Eye-Movement Cross-correlogram method originally introduced by Mulligan and colleagues (Mulligan et al., 2013). It constitutes an extension of it to the clinical domain. The spatio-temporal properties of eye movements are a collection of features extracted from the continuous gaze tracking of a stimulus. The stimulus trajectory is designed to keep the observers engaged, minimize learning effect and induce saccadic movements of different magnitude. All these characteristics are desirable in a test that aims to detect clinically relevant oculomotor abnormalities.

Some of the derived spatio-temporal features of eye movements are more sensitive to physical changes in the stimuli (e.g. speed, contrast) while others are more sensitive to the state of the observer (e.g. underlying clinical condition). Taken together they quantify the performance of an observer's visual system in a dynamic context. Noticeably, they do not correlate with static functional measures such as visual acuity and contrast sensitivity.

2 Algorithm description

2.1 Schematic overview

This section shows an overview of the process necessary to evaluate the spatio-temporal properties of eye movement. Figure S1 summarizes the steps necessary for the data acquisition, while Figure S2 summarizes the feature extraction process.

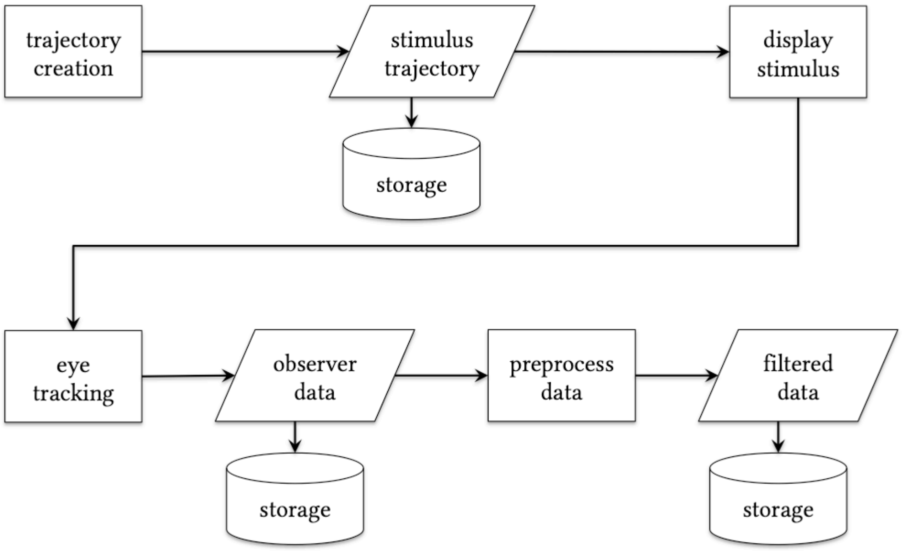

Figure S1. Schematic representation of the data acquisition process.

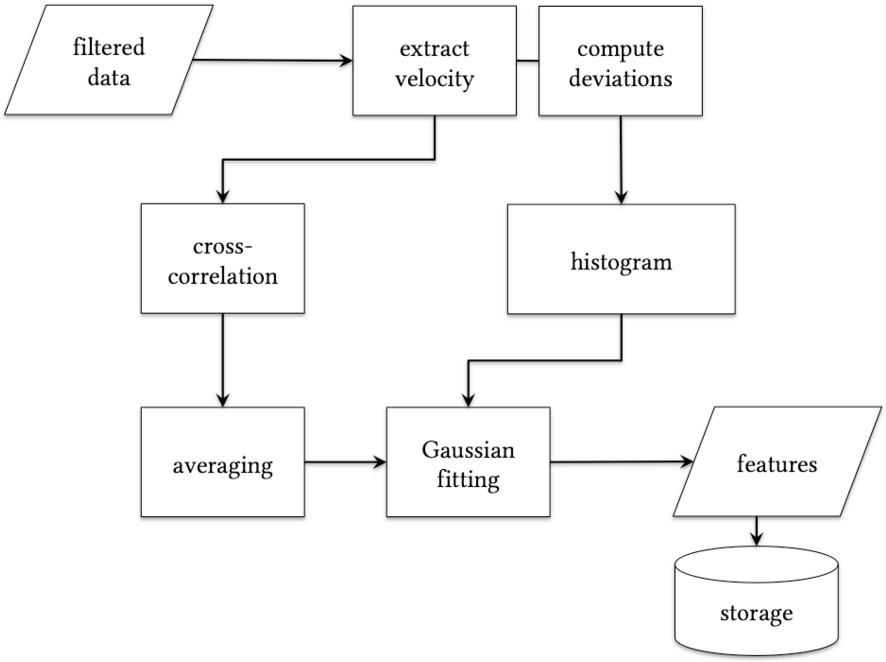

Figure S2. Schematic representation of the feature extraction process.

2.2 Stimulus properties

2.2.1 Stimulus visualization

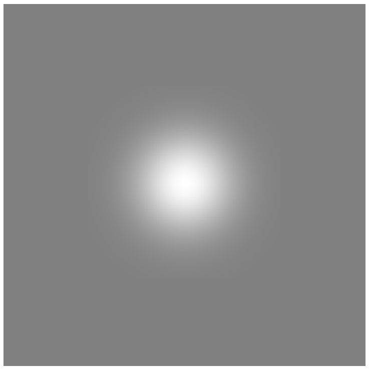

Figure S3. An example of a Gaussian luminance blob used as a moving stimulus.

2.2.2 Properties of the stimulus trajectory

The stimulus trajectory consists of a constrained, random path. The two constraints are: (1) the stimulus trajectory must stay within the boundaries of the screen. (2) The stimulus trajectory cannot contain periodic autocorrelations.

The stimulus trajectory is constructed by generating an array of velocity values where, at each time-point, the velocity values for the horizontal and vertical components are drawn from a Gaussian distribution. This distribution is always zero-centered, and its standard deviation can be adjusted to modulate the final velocity of the stimulus. The values used in this study are sigma = ~ 64 deg/sec for the horizontal component and ~32.33 deg/sec for the vertical component. These values have been chosen empirically, to fit the screen's aspect ratio and to produce a stimulus sufficiently hard to follow for healthy observers while challenging, yet not impossible to follow, for visually impaired observers.

The velocity vector is low-pass filtered (cut-off = 10 Hz) by convolution with a Gaussian kernel such that excessive jitter is minimized. Subsequently, via temporal integration, velocities are transformed into positions of the stimulus $s(t) = \begin{bmatrix} s_x \\ s_y \end{bmatrix}$. In order to induce the observer to also perform saccadic movements in addition to the smooth pursuit, we created trajectories with random stimulus displacements. This is achieved by randomly juxtaposing epochs of 2 seconds each (Figure S4) taken from the original 6 trajectories.

During a typical assessment, each observer is presented with 6 different trajectories of 20 seconds each per pursuit modality, one being with and the other without saccadic insertion, subsequently referred to as *smooth* and *saccadic pursuit* conditions, respectively.

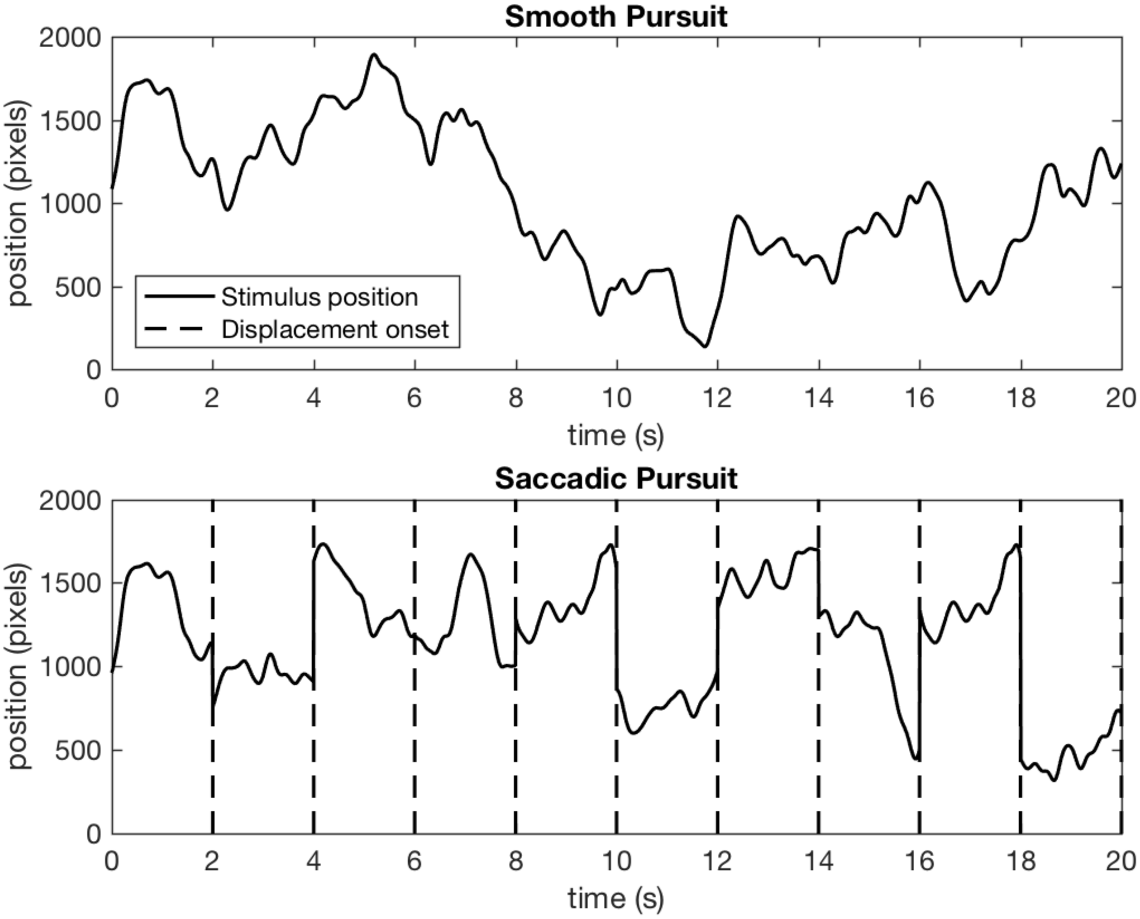

Figure S4. Examples of the stimulus trajectory (horizontal component) over time for *smooth* and *saccadic* pursuit.

2.3 Pre-processing of eye-tracking data

The data acquired consists of time series of eye gaze positions $p(t) = \begin{bmatrix} p_x \\ p_y \end{bmatrix}$ expressed in visual field coordinates.

Blinks and other artifacts are removed as follows: blink periods are identified by spikes in the vertical gaze velocity (first derivative of p_y > 300 deg/sec) followed by a plateau (first derivative of $p_y = 0$) or missing data. This specific artifact is caused by how video-based eye-trackers compute gaze position: when the eyelid is closing due to blinking, it partially covers the pupil, which is erroneously interpreted as a rapid shifting upwards (Figure S5-A). The closed eye is then recorded as missing data or as the last valid position recorded. Each blink period found is dilated by 5 samples on both sides. If the total data loss (due to blinks or otherwise) exceeds 25% of the trial duration, the entire trial is removed from further analysis. Lastly, the data in the blink-period is imputed by fitting an autoregressive model (Akaike, 1969) using 10 samples preceding and following each of the above-defined blank periods (Figure S5-B). After all blinks are removed and missing data are filled, a Butterworth low-pass filter (half-power frequency = 0.5 Hz) applied to *p(t)* is used to remove any

instrument noise from the recorded gaze positions. An example of time-series pre- and post-processed with this "blink-filtering algorithm" is shown in Figure S5-C.

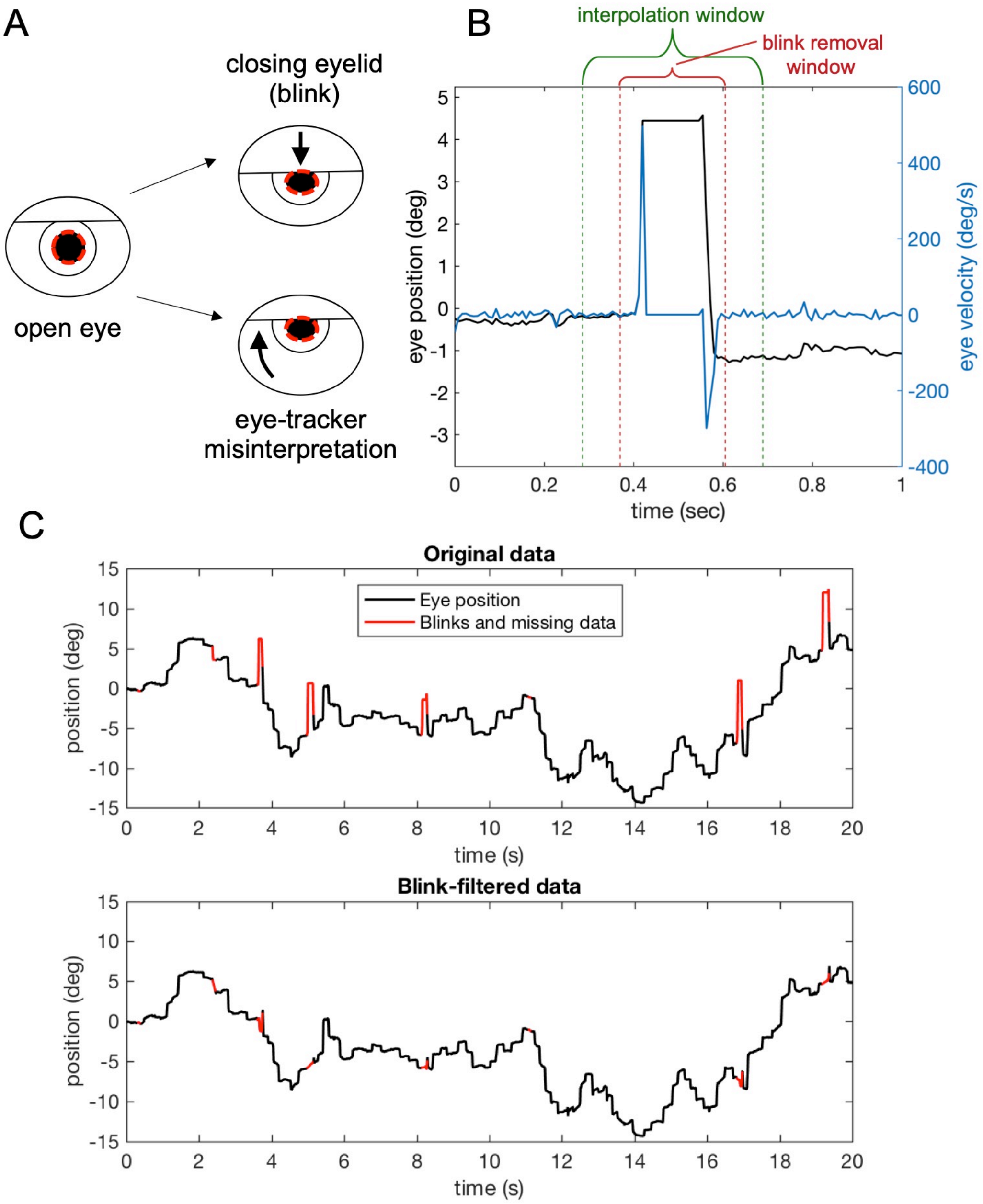

Figure S5. A. Schematic representation of the eye-tracker gaze misinterpretation. When the eyelid partially occludes the pupil during a blink, the eye-tracker erroneously interprets the shortened pupil as being vertically displaced. **B.** Detail of a blink artifact. The red lines show the temporal window within which the data is removed, while the green lines show the temporal windows from which the data is pooled in order to interpolate the missing part. **C.** Example of a time-series before and after applying the blink-filtering algorithm.

2.4 Spatio-temporal features extraction

This section describes how temporal, spatial and spatio-temporal features are extracted from the data. The parameters that reflect primarily the temporal aspects of the oculomotor behavior, such as response delay and velocity oscillations, are referred to as "temporal" features. The parameters that reflect the spatial aspects of the observer's performance, like accuracy, are referred as "spatial" features. The "spatio-temporal" category contains the remaining parameters (here called *observation noise variance* and *cosine dissimilarity*) that are affected by both temporal delays and spatial inaccuracies.

2.4.1 Temporal features

The post-processed time-series of gaze positions $p(t)$ and stimulus positions $s(t)$ are transformed into their respective velocities $v_p(t)$ and $v_s(t)$ by taking their first-order temporal derivative.

A normalized time-shifted cross-correlation is applied between $v_p(t)$ and $v_s(t)$ separately for the horizontal and vertical components (Figure S6-A shows an example of $v_p(t)$ and $v_s(t)$, horizontal component). The time-shift ranges from -1 to +1 sec with a step size of 1 inter-frame interval, which depends on the apparatus in use. Each of the 6 data-acquisitions of 20 seconds leads to two cross-correlograms, one for the horizontal component and one for the vertical. The 6 resulting cross-correlograms of each component are then averaged and the resulting averaged cross-correlogram (CCG, see Figure S6-B) is fitted with a Gaussian model, which returns the following parameters: *amplitude*, *mean* (μ), *standard deviation* (σ) and *variance explained* (R^2). These parameters constitute the group of temporal features, a detailed description will follow in the section "Properties of spatio-temporal features").

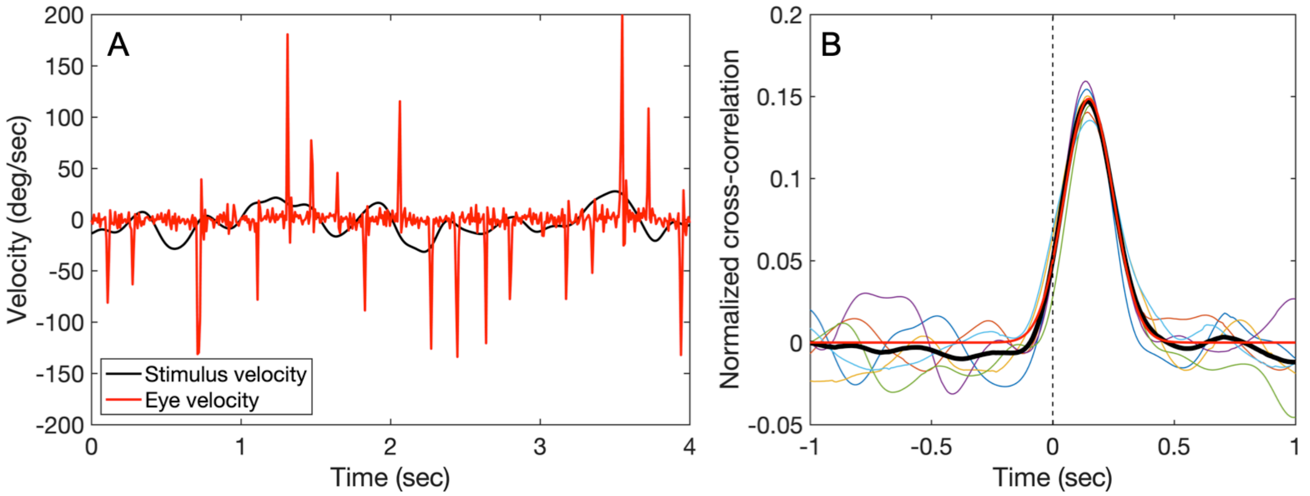

Figure S6. A. Example of ocular horizontal velocity in response to the tracking target. **B.** Example of a CCG resulting from the average of the 6 individual cross-correlograms obtained after each tracking trial. Black line shows the average CCG, red line shows the fitted Gaussian model, the remaining colored lines show the individual cross-correlograms.

2.4.2 Spatial features

An array positional deviation between the stimulus and the eye $d(t) = \begin{bmatrix} d_x \\ d_y \end{bmatrix}$ is computed for each time-point t in *p(t)* and *s(t)* as $d_x = p_x - s_x$ and $d_y = p_y - s_y$ (Figure S7-A shows an example of *p(t)*, *s(t)* and *d(t)*, horizontal component). Next, the resulting 6 arrays $d(t)_{1...6}$ are concatenated (*N.B.: not averaged*) and a probability density distribution (PDD) is drawn from the resulting concatenated array (Figure S7-B). A Gaussian model is fitted to the PDD and, analogously to the *temporal features*, the parameters obtained are *amplitude*, *mean* (μ), *standard deviation* (σ) and *variance explained* (R^2). These parameters constitute the group of spatial features; a detailed description will follow in the section "*Properties of spatio-temporal features*").

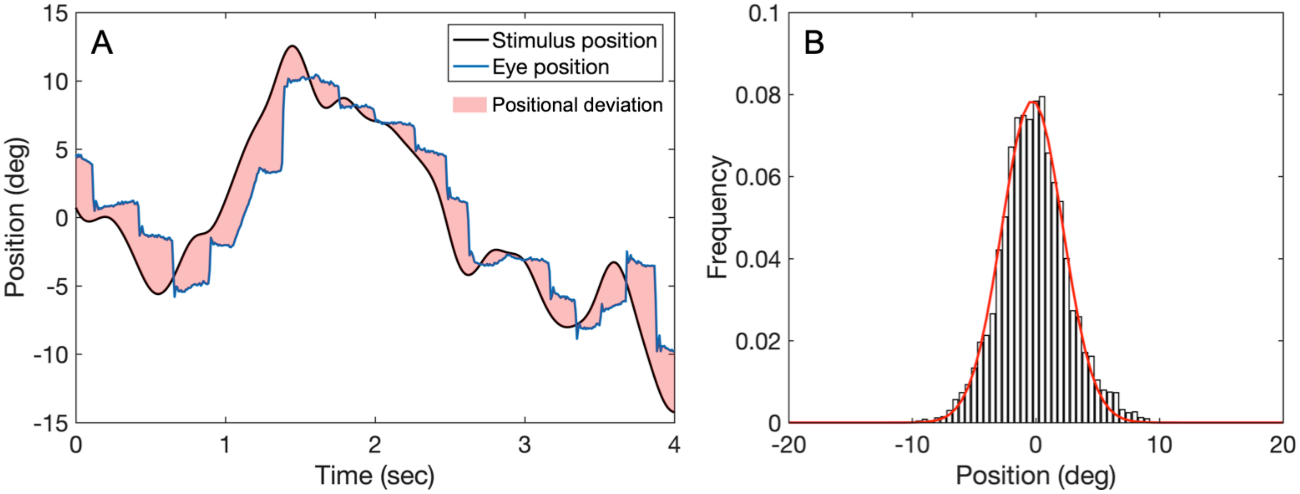

Figure S7. A. Example of ocular horizontal position in response to the tracking target. The deviations between stimulus and eye position are aggregated for all trials. **B.** Example of PDD resulting from the histogram of the aggregated positional deviations. The red line shows the fitted Gaussian model.

2.4.3 Spatio-temporal features

Observation noise variance: to compute this parameter, continuous tracking behavior is modeled by dynamic linear systems, with their solutions being provided by state-space models such as the Kalman filter (Bonnen et al., 2015).

An example of these linear systems, as reported by Huk and colleagues (Huk et al., 2018), is as follows:

$$x_t = F_t x_{t-1} + w_t;\ w_t \sim N(0, Q_t)$$

$$y_t = H_t x_{t-1} + v_t;\ v_t \sim N(0, R_t)$$

where x_t is the stimulus parameter tracked by the observer at time *t* (e.g., the coordinates of a moving target), F_t is the process transition matrix, w_t is the process noise, y_t is the noisy internal response (e.g., a pursuit eye movement), H_t is the observation model that maps the true state space to the observation space, and v_t is the internal noise. Assuming that both the process noise (related to the

stimulus) and the internal noise (related to the observer) are Gaussian, the Kalman filter provides the following estimators:

$$\hat{x}_{t|t-1} = F_t \hat{x}_{t-1}$$

$$\hat{x}_t = \hat{x}_{t|t-1} + K_t(y_t - H_t \hat{x}_{t|t-1})$$

where $\hat{x}_t$ is the estimate of x_t; $\hat{x}_{t|t-1}$ is the estimate of x_t given all the information up to but not including the current time step, t; and K_t is the Kalman gain, which is calculated from estimates of the covariance (i.e., an estimate of the level of uncertainty in the system). A Kalman filter typically provides an estimate of the current unknown state of a system for which some structural properties are known, such as system noise and observer noise (w_t and v_t, respectively) and state-transition matrices (F_t and H_t, respectively).

In our context, however, the "unknown state of the system" is not unknown at all: it is the recorded position of the gaze in response to the motion of the target at a given time. Therefore, starting from the gaze position in response to the moving target, it is possible to estimate the observation noise variance, which reflects the overall noisiness of the observer.

To do so, we reversed the Kalman filter application as described by Bonnen and colleagues (Bonnen et al., 2015), while also assuming that the observation model that maps the true state space to the observation space H_t is equal to 1 (i.e. assuming that the oculomotor system is a simple linear system without nonlinear dynamics).

When the observation noise variance is low relative to the target displacement variance (i.e., target visibility is high), the difference between the previous position estimate and the current noisy observation is likely to be due to changes in the position of the target. That is, the observation is likely to provide reliable information about the target position. As a result, the previous estimate will be given little weight compared to the current observation. Tracking performance will be fast and have a short lag. On the other hand, if the observation noise variance is high relative to the target displacement variance (i.e., target visibility is low), then the difference between the previous position estimate and the current noisy observation is likely driven by observation noise. In this scenario, little weight will be given to the current observation while greater weight will be placed on the previous estimate. Tracking performance will be slow and have a long lag (Bonnen et al., 2015).

Dissimilarity: this parameter entails a measure of dissimilarity between the two vectors (A) stimulus positions and (B) gaze position. In the context of comparing tracking coordinates, the cosine similarity of two positional vectors is bounded between 0 and 1, therefore the dissimilarity is computed as the inverse of the cosine similarity:

$$1 - \frac{\sum_i^n A_i \, B_i}{\sqrt{\sum_i^n A_i^2} \sqrt{\sum_i^n B_i^2}}$$

It has the useful property of being unaffected by the length of the vectors. Since it is computationally inexpensive, it is a useful feature to evaluate the performance of an observer in real-time. In healthy observers, it usually correlates strongly with the *Observation noise variance*.

3 Properties of the spatio-temporal features

Table S1 provides details about each spatio-temporal feature:

Feature Name	*Description*	*Value Range*
F1: CCG amplitude	Maximum correlation between stimulus and eye velocities.<br>Higher values → better performance	[-1 1]
F2: CCG mean	Lag between stimulus and eye (in ms).<br>Lower values → better performance	[0 ∞]
F3: CCG standard deviation	Temporal uncertainty: window of temporal integration that the observer needs in order to track the stimulus (in ms).<br>Lower values → better performance	[0 ∞]
F4: CCG variance explained	Consistency of tracking performance across trials.<br>Higher values → better performance	[0 1]
F5: PDD amplitude	Most frequent positional deviation.<br>Higher values → better performance	[0 1]
F6: PDD mean	Spatial bias.<br>Lower values → better performance	[0 ∞]
F7: PDD standard deviation	Positional uncertainty: spread of the positional deviations.<br>Lower values → better performance	[0 ∞]
F8: PDD variance explained	Normality of the positional deviation distribution.<br>Higher values → better performance	[0 1]
F9: Observation noise variance	Sensory noise estimated by measuring the variance of the observational noise with a Kalman filter(Bonnen et al., 2015).<br>Lower values → better performance	[0 ∞]
F10: Dissimilarity	Inverse of cosine similarity between gaze and stimulus vectors of positions.<br>Lower values → better performance	[0 1]

Table S1. Name and details of the spatio-temporal features used to quantify the observer's tracking performance. Each feature is computed separately for the horizontal and vertical components of the eye movements (CCG: cross-correlogram; PDD: positional deviations distribution).

Together, all these features constitute our feature-space. An overview of the correlations normally present in the feature-space is shown in Figure S8. This example is built using the data from our control group of participants. In a healthy population, certain features highly correlate (or anti-correlate) amongst each other or between their respective horizontal and vertical counterparts. Usually, highly correlated features within a dataset are not particularly useful (as they provide redundant information). However, the presence or absence of expected correlations in a group of observers could provide valuable insights. A noticeable example is feature F4 (variance explained of the gaussian fit to the CCG). By itself, this feature is very uninformative in the healthy population: during *smooth pursuit* condition it does not correlate with any other feature (see Figure 8-A, left, lines 4 and 14) as it often shows a ceiling-effect (see Figure S8-B, panel F4, all values are above 0.90). However, the introduction of saccadic displacements removes the ceiling-effect in the vertical component (see Figure S8-C, panel F4, y axis) and increases the correlations with other features (Figure S8-A, right, line 14). This peculiar behavior makes this feature an excellent anomaly detector when testing different populations.

On the other hand, features such as F2 show very consistent correlations between the horizontal and vertical components and with other features as well. It is thus suitable for measuring performance also in a within-subject context.

Overall, all spatio-temporal features contribute to creating a unique “oculomotor fingerprint” of the observer who performed the test, which in turn can be used as a powerful, yet simple, screening tool.

Lastly, in healthy controls, the spatio-temporal features of eye-movements are independent from other measures of visual function, such as visual acuity and contrast sensitivity (see Figure S9). The cumulative histogram of the Spearman’s rank coefficients is not different from that of the null hypothesis, which was obtained by randomizing the correlation matrix and calculating the 95% confidence intervals with a permutation test. Therefore, we conclude that neither visual acuity (VA) nor contrast sensitivity (CS) is correlated with any of the spatio-temporal properties measured with continuous tracking, both for the *smooth pursuit* and *saccadic pursuit* modalities.

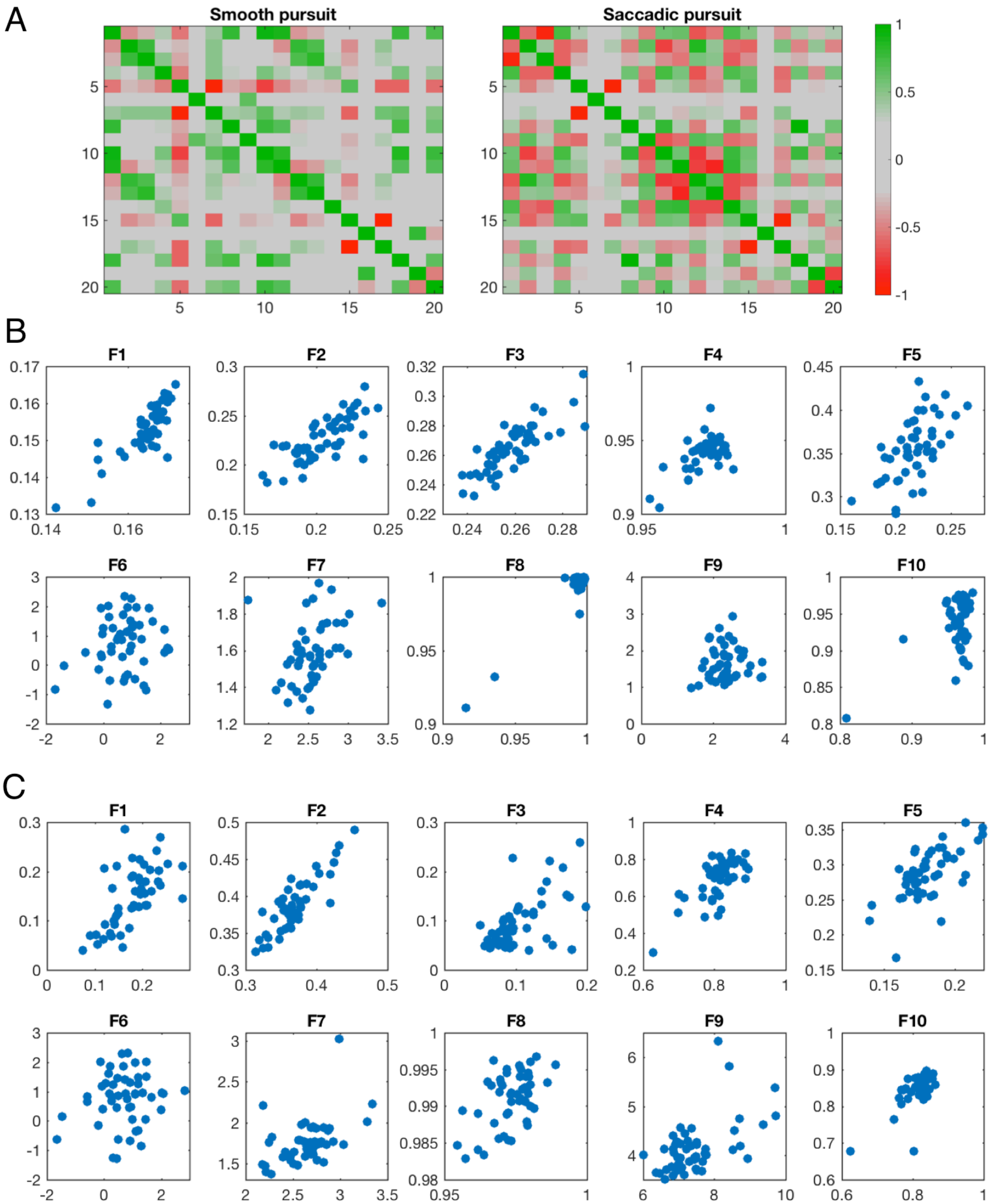

Figure S8. A. Correlation matrices between all spatio-temporal features. **B.** Correlations (or lack thereof) between horizontal and vertical components of each spatio-temporal feature obtained during smooth pursuit tracking. **C.** Same as B, but for feature values obtained during saccadic pursuit tracking.

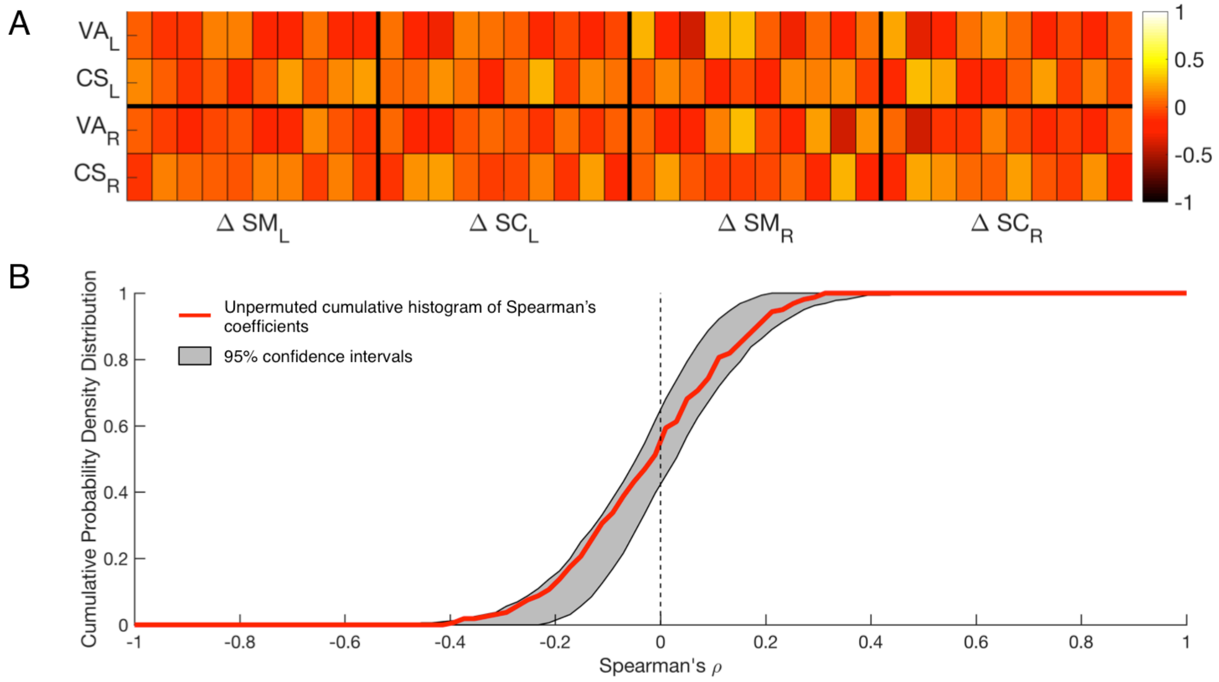

Figure S9. Correlation matrix between spatio-temporal features and visual functions. VA = visual acuity, CS = contrast sensitivity, SM = smooth pursuit, SC = saccadic pursuit, L = left eye, R = right eye.

4 Supplementary references

Akaike, H. (1969). Fitting autoregressive models for prediction. In *Annals of the Institute of Statistical Mathematics* (Vol. 21, Issue 1, pp. 243–247). https://doi.org/10.1007/bf02532251

Bonnen, K., Burge, J., Yates, J., Pillow, J., & Cormack, L. K. (2015). Continuous psychophysics: Target-tracking to measure visual sensitivity. *Journal of Vision*, *15*(3). https://doi.org/10.1167/15.3.14

Huk, A., Bonnen, K., & He, B. J. (2018). Beyond Trial-Based Paradigms: Continuous Behavior, Ongoing Neural Activity, and Natural Stimuli. *Journal of Neuroscience*, *38*(35), 7551–7558.

Mulligan, J. B., Stevenson, S. B., & Cormack, L. K. (2013). Reflexive and voluntary control of smooth eye movements. In *Human Vision and Electronic Imaging XVIII*. https://doi.org/10.1117/12.2010333
